# Enhanced thermoelectric performance of SnSe by controlled vacancy population

**DOI:** 10.1186/s40580-023-00381-7

**Published:** 2023-07-07

**Authors:** Ji-Eun Lee, Kyoo Kim, Van Quang Nguyen, Jinwoong Hwang, Jonathan D. Denlinger, Byung Il Min, Sunglae Cho, Hyejin Ryu, Choongyu Hwang, Sung-Kwan Mo

**Affiliations:** 1 Advanced Light Source, Lawrence Berkeley National Laboratory, Berkeley, CA 94720 USA; 2 Max Planck-POSTECH/Hsinchu Center for Complex Phase Materials, Max Plank POSTECH/Korea Research Initiative (MPK), Gyeongbuk, 37673 South Korea; 3 Center for Spintronics, Korea Institute of Science and Technology, Seoul, 02792 South Korea; 4 Department of Physics, Pusan National University, Busan, 46241 South Korea; 5 Korea Atomic Energy Research Institute, Daejeon, 34057 South Korea; 6 Department of Physics, Pohang University of Science and Technology (POSTECH), Pohang, 37673 South Korea; 7 Department of Physics and Energy Harvest-Storage Research Center, University of Ulsan, Ulsan, 44610 South Korea; 8 Department of Physics, Kangwon National University, Chuncheon, 24341 South Korea

**Keywords:** Thermoelectric, Defect engineering, Electron band structure, Vacancy, SnSe

## Abstract

The thermoelectric performance of SnSe strongly depends on its low-energy electron band structure that provides high density of states in a narrow energy window due to the multi-valley valence band maximum (VBM). Angle-resolved photoemission spectroscopy measurements, in conjunction with first-principles calculations, reveal that the binding energy of the VBM of SnSe is tuned by the population of Sn vacancy, which is determined by the cooling rate during the sample growth. The VBM shift follows precisely the behavior of the thermoelectric power factor, while the effective mass is barely modified upon changing the population of Sn vacancies. These findings indicate that the low-energy electron band structure is closely correlated with the high thermoelectric performance of hole-doped SnSe, providing a viable route toward engineering the intrinsic defect-induced thermoelectric performance via the sample growth condition without an additional *ex-situ* process.

**Graphical Abstract:**

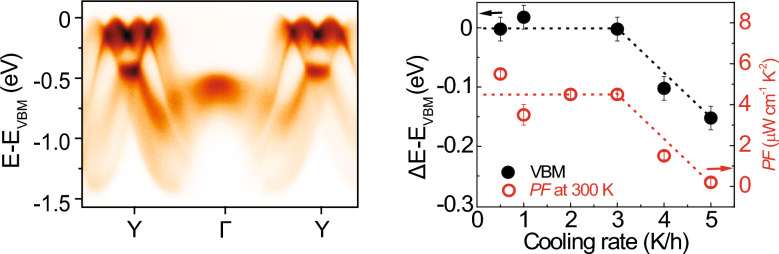

**Supplementary Information:**

The online version contains supplementary material available at 10.1186/s40580-023-00381-7.

## Introduction

Thermoelectric materials are of great importance for renewable energy technology since they can generate electricity from the temperature gradient of wasted heat [1–4]. The quantity of interest that determines the thermoelectric performance is a dimensionless figure of merit *ZT* = *S*^*2*^*σT/κ*, which is influenced by the electronic and phononic properties of the thermoelectric material. Here *S*, *σ*,* T*, and *κ* are the Seebeck coefficient, electric conductivity, temperature, and thermal conductivity, respectively, while *S*^*2*^*σ* is called a power factor (*PF*)*.* One of the ultimate goals in the research of thermoelectric materials is to achieve a high *ZT* value, which is generally considered a challenging task because the physical parameters comprising *ZT* are correlated and often act adversely on each other [1, 4] One effective strategy to optimize both the electrical and thermal properties is defect engineering, which can simultaneously increase the charge carrier concentration and reduce the lattice thermal conductivity in the material [5–8]. The electronic contribution to the thermoelectric performance that is mainly characterized by *PF*, however, is directly understood by the electron band structure, while the phonon dispersion provides essential information on the phononic contribution.

SnSe is one of the remarkable van der Waals materials with great potential for applications [9, 10]. It has attracted recent research interest owing to its high *ZT* value, due to the ultrahigh *PF*, which is attributed to a multi-valley valence band maximum (VBM) [11–15] and band renormalization, i.e., change in the effective mass of the multi-valley VBM [11–15], in conjunction with low thermal conductivity attributed to anisotropic and anharmonic phonon dispersions [16, 17]. However, while the multi-valley VBM was recently observed through angle-resolved photoemission spectroscopy (ARPES) studies [18–21], the correlation between the electron band structure and the thermoelectric performance has not been well understood.

In this article, we report a systematic study on the evolution of the electron band structure of a SnSe single crystal with a controlled hole carrier density, investigated using ARPES measurements and first-principles density functional theory (DFT) calculations. The *PF* value of SnSe depends on the cooling rate during its growth process that determines the population of Sn vacancy [22]. ARPES data reveal that the slower cooling rate also leads to the overall shift of the multi-valley VBM towards the lower binding energy, indicating hole doping of SnSe. The VBM shift exactly follows the behavior of the *PF* value [22]. Our study reveals the role of the low-energy electron band structure of SnSe on its thermoelectric performance that is tuned by the hole-carrier density.

## Methods

Single crystal SnSe was synthesized with several different cooling rates as described elsewhere [22]. ARPES measurements were performed at the Beamlines 4.0.3 and 10.0.1 of the Advanced Light Source (ALS), Lawrence Berkeley National Laboratory. Single crystal SnSe was cleaved and measured in an ultra-high vacuum with a base pressure of 4 × 10^–11^ Torr. The measurements were done at 20 K using photon energies of 72 eV and 60 eV. Prior to the ARPES measurement, the charging effect has been examined for all the samples (see Additional file 1). The energy and momentum resolutions were set to be 18 meV and 0.01 Å^-1^, respectively. The electron band structure of SnSe was calculated utilizing the DFT implemented in full-potential linearized augmented plane wave package [23], within Perdew-Burke-Ernzerhof functional combined with modified Becke-Johnson potential (PBE + mBJ) [24]. The coherent potential approximation (CPA) within Korringa-Kohn-Rostoker (KKR) Green’s function method implemented in SPR-KKR package [25] was adopted to understand the role of Sn vacancies on the electron band structure. For better determination of the Fermi energy, the Lloyd formula has been used [26].

## Results and discussion

### Electron band strucutres of SnSe

**Fig. 1:**
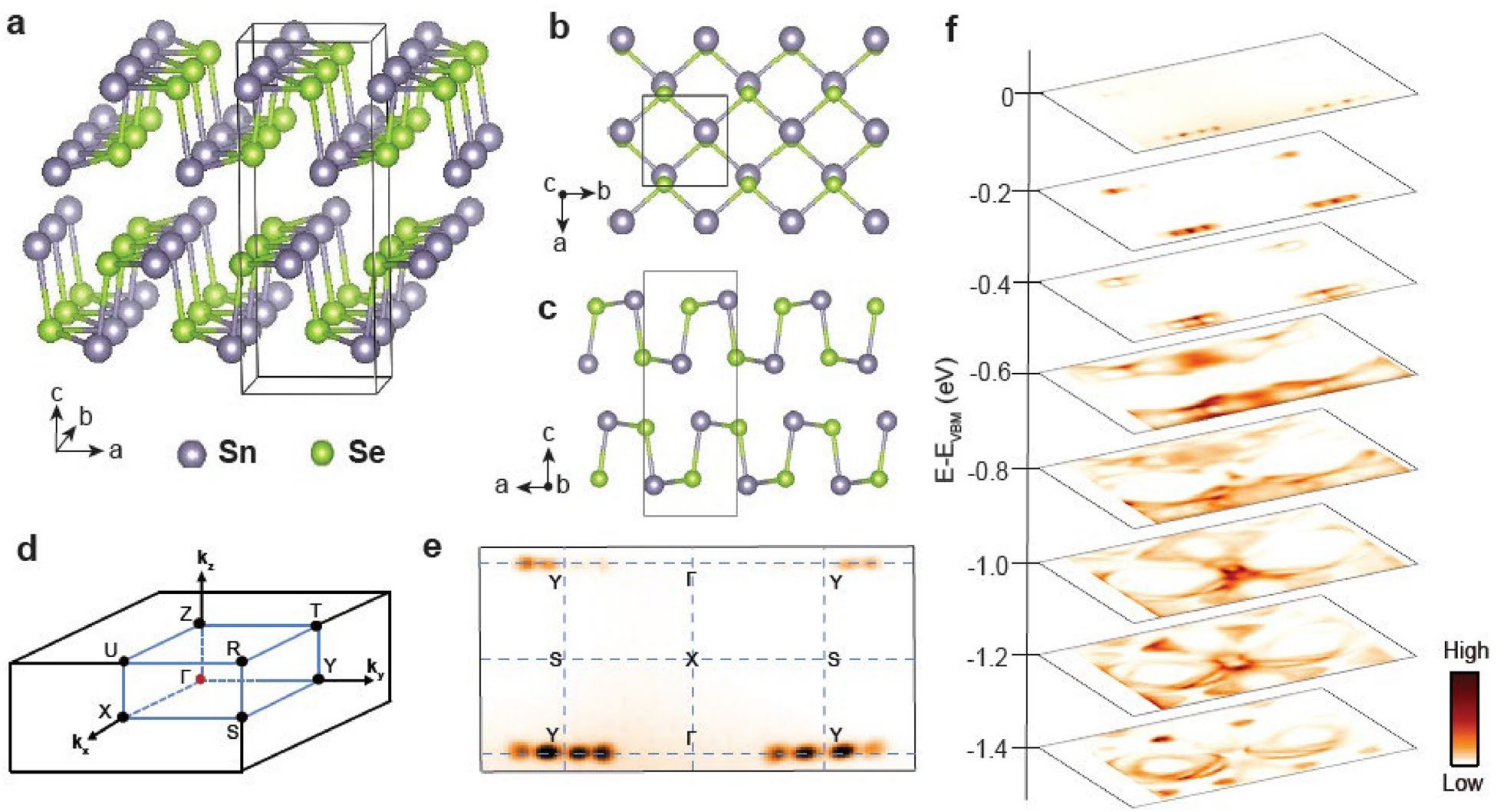
The crystal structure and ARPES data of SnSe. **a**–**c** The crystal structure of SnSe (**a**) with a top (**b**) and side views (**c**). The black lines indicate the conventional unit cell of SnSe. **d** A Brillouin zone of the *Pnma* phase. **e** A constant energy ARPES intensity map taken at VBM. **f** Constant energy ARPES intensity maps taken at several different *E*–*E*_VBM_ from − 1.4 eV to 0 eV

The crystal structure of SnSe belongs to the space group *Pnma* with lattice constants *a* = 4.15 Å, *b* = 4.44 Å, and *c* = 11.57 Å, as shown in Fig. 1a–c [27]. Two layers of SnSe are stacked along the *c*-direction with the weak van der Waals interaction [28], constituting the SnSe unit cell depicted in Fig. 1a. Figure 1e shows a constant energy ARPES intensity map taken at the energy corresponding to VBM (*E*_VBM_), since SnSe is an insulator with a band gap of 0.8–0.9 eV [11, 15, 29]. Equally-spaced four dots are observed around the Y point that expand to larger-sized circles at higher binding energy, as shown in Fig. 1f, indicating that these bands are hole bands.

**Fig. 2:**
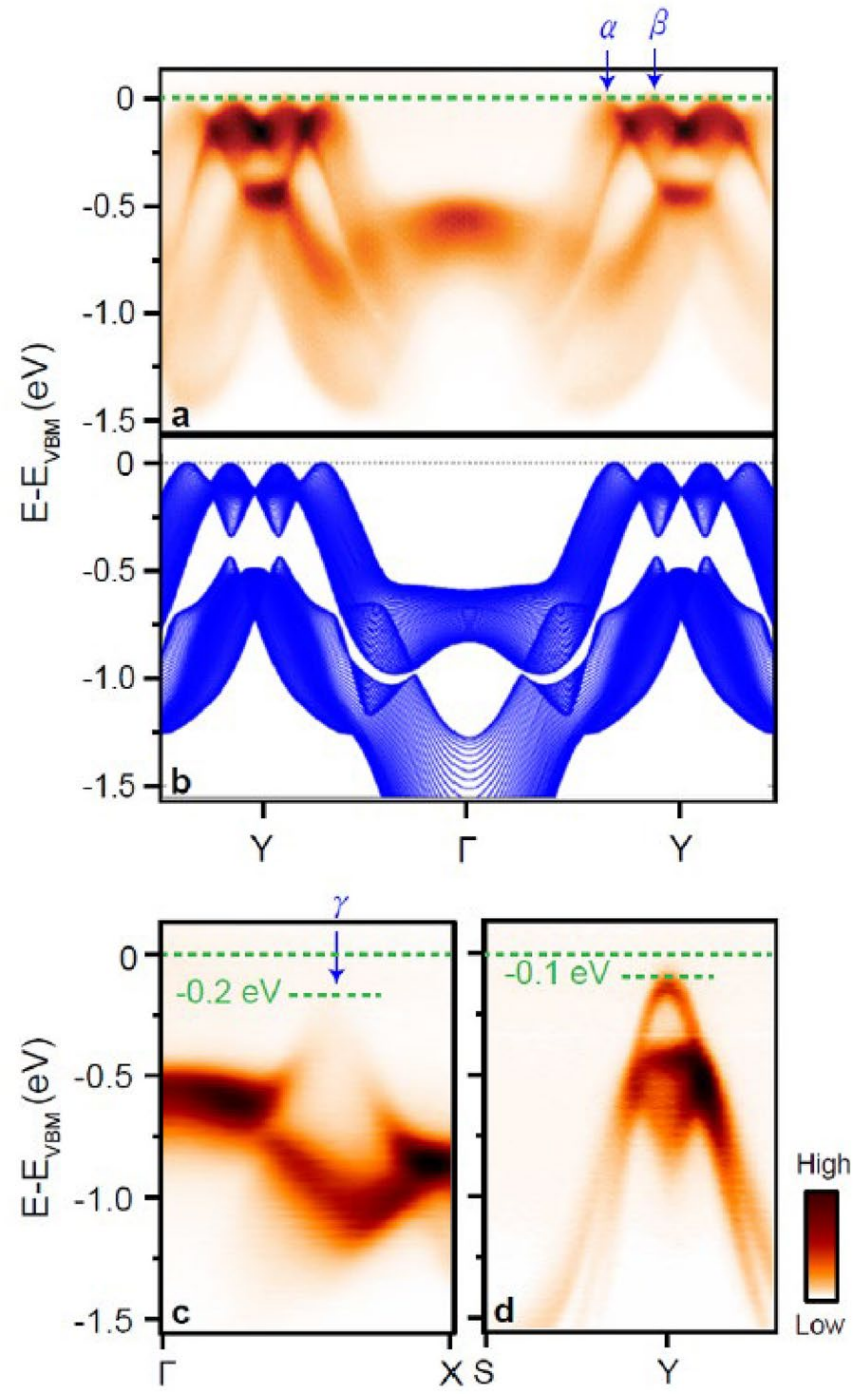
ARPES spectra and theoretical calculations. **a** ARPES spectra of a SnSe single crystal with a crystal growth cooling rate of 0.5 K/h taken along the Y-Γ-Y direction. **b** The calculated electron band structure of SnSe along the Y-Γ-Y direction. **c**, **d** ARPES spectra taken along the Γ-X (**c**) and S-Y (**d**) directions

Figure 2 shows the low-energy electron band structure of SnSe. Figure 2a is the electron band structure measured by ARPES along the Y-Γ-Y direction in the Brillouin zone of the SnSe (Fig. 1d, e). Around the Y point, four humps and three dips are observed close to *E*_VBM_. Two of the humps in the first Brillouin zone are denoted by *α* and *β*. To understand the observed ARPES spectra, the electron band structure of SnSe was calculated using the PBE + mBJ method, as shown in Fig. 2b. The observed ARPES spectra are in agreement with the calculated electron band structure, showing the characteristic four humps around the Y point. The calculated energy gap is 0.86 eV, which is consistent with previous results [11, 15, 29], indicating that the PBE + mBJ method well describes the electronic correlations in SnSe. The difference between the measured and calculated electron band structure is the lack of photoelectron intensity at 1.0 ~ 1.5 eV below *E*_VBM_ around the Γ point and the bandwidth of the low-energy electron band structure. While the former is attributed to the matrix element effect, the decrease of the bandwidth by 17% in the calculated band structure (compared at the bottom of the band at half the unit cell, i.e., ½ΓY) might originate from the slight difference in the structural parameters [18, 19].

Along the Γ-X direction, another hole band denoted by *γ* is observed with its top at 0.2 eV below *E*_VMB_ as shown in Fig. 2c. Along the Y-S direction, the top of the hole band is observed at 0.1 eV below *E*_VBM_, indicating that the energy difference between the hump and dip observed close to *E*_VMB_ is 0.1 eV as shown in Fig. 2c. The observed ARPES spectra are consistent with previously reported results taken at *k*_z_ = 16.8(π/c) [18], corresponding to a photon energy of 72 eV that has been also used in our experiments.

### Engineering thermoelectric performance

**Fig. 3:**
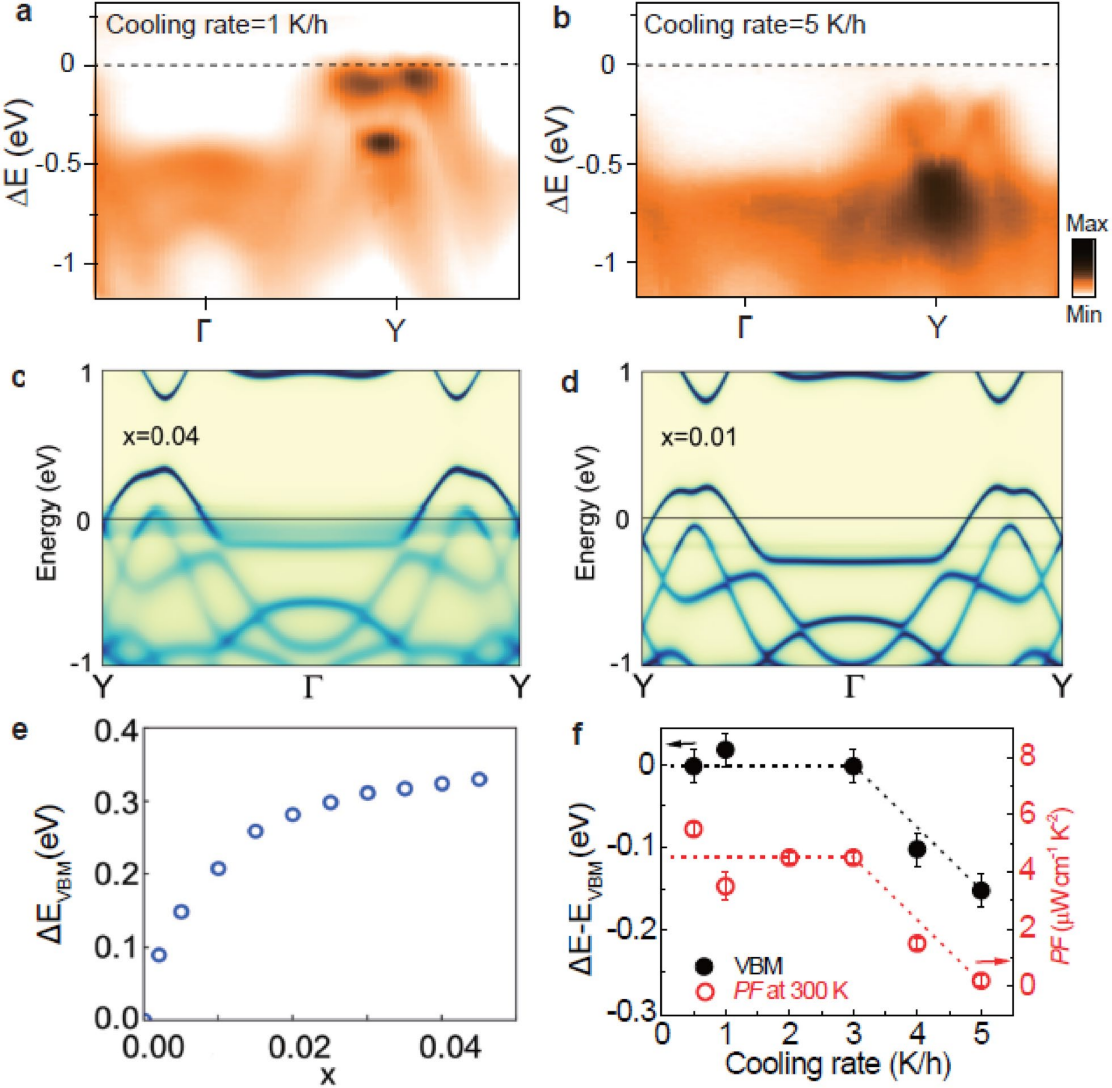
Relation between *PF* and the electron band structure. **a**, **b** ARPES intensity maps taken along the Γ-Y direction of SnSe with a crystal growth cooling rate of 1 K/h (**a**) and 5 K/h (**b**). Δ*E* is the energy relative to the VBM of the 0.5 K/h sample. **c**, **d** A calculated electron band structure along the Γ-Y-Γ direction using the KKR-CPA method for Sn_1-*x*_Se with *x* = 0.04 (**c**) and *x* = 0.01 (**d**). **e** Doping dependence of the VBM obtained by calculations. **f** Crystal growth cooling rate dependence of Δ*E–E*_VBM_ (closed circles) and *PF* (open circles) extracted from the ARPES data and previous transport results [22], respectively

In order to study the effect of the cooling rate during SnSe single crystal growth on the electron band structure, Fig. 3a, b show ARPES spectra measured along the Y-Γ-Y direction for two representative SnSe crystals grown with different cooling rates. The dashed line denotes the *E*_VBM_ of the data shown in Fig. 2a, corresponding to a cooling rate of 0.5 K/h. At 1 K/h, *E*_VBM_ remains almost the same as that of 0.5 K/h. However, with increasing cooling rate to 5 K/h, the overall multi-valley band shifts toward higher binding energy, indicating that the cooling rate tunes the charge carrier density of SnSe. Since the cooling rate during the growth process determines the population of the Sn vacancy, as shown in recent transport and STM study [22], the electron band structures have been calculated for different Sn vacancy levels using the KKR-CPA method (Fig. 3c, d). Here, *x* denotes the population of Sn vacancies compared to the number of Sn atoms in a perfect SnSe crystal, implying that the stoichiometry of the sample is Sn_1–x_Se. With increasing *x*, the overall multi-valley VBM shifts toward lower binding energy. The non-dispersive fuzzy states that become obvious at *x* = 0.04 originate from impurity scattering. The shift of *E*_VBM_ as a function of doping ratio *x*, estimated from the shift of the VBM spectral weight in KKR calculation, is summarized in Fig. 3e. With increasing *x*, Δ*E*_VBM_ gradually increases, indicating that Sn vacancies lead to hole doping of SnSe. Based on the comparison, the shift of *E*_VBM_ with the increasing cooling rate shown in Fig. 3a, b indicates the decreasing population of Sn vacancies. As a result, a lower cooling rate generates higher hole doping of SnSe.

Figure 3f shows the cooling rate dependence of *E*_VBM_ (black filled circles), compared to that of *PF* [22] (red empty circles). *E*_VBM_ does not show a notable change up to a cooling rate of 3K/h. When the cooling rate further increases, *E*_VBM_ decreases, indicating that SnSe gradually has less hole doping due to Sn vacancies. Surprisingly, Δ*E*_VBM_ exactly follows the cooling rate dependence of *PF* taken at 300K [22]. This finding suggests that the high *PF*, observed in hole-doped SnSe [11–15], is closely correlated with the charge carrier density (hole concentrations), i.e., the population of Sn vacancies, which is determined by the cooling rate during the single crystal growth of SnSe.

An alternative origin of the high *PF* value observed in hole-doped SnSe [11–15] is the light effective mass of the multiple hole bands, i.e., *α*, *β*, and *γ* bands, which tends to strongly influence the electric properties of a material. Indeed, parabolic fitting to the top of the *α*, *β*, and *γ* bands shown in Fig. 2 corresponding to a cooling rate of 0.5 K/h confirms the light effective mass of m^*α^_ΓX_ = 0.19 ± 0.02 *m*_*e*_ and m^*α^_ΓY_ = 0.11 ± 0.01 *m*_*e*_ for the *α* band, m^*β^_ΓX_ = 0.15 ± 0.01 *m*_*e*_ and m^*β^_ΓY_ = 0.21 ± 0.02 *m*_*e*_ for the *β* band, and m^*γ^_ΓX_ = 0.08 ± 0.02 *m*_*e*_ and m^*γ^_ΓY_ = 0.09 ± 0.02 *m*_*e*_ for the *γ* band, when *m*_*e*_, m^*^_ΓY_, and m^*^_ΓX_ are free electron mass, when the effective mass of each band estimated parallel and perpendicular to the Γ-Y direction, respectively. The effective masses obtained from ARPES and band structure calculations are consistent with the previous results [18, 19]. At a faster cooling rate of 5 K/h, the effective masses of the *α* and *β* bands along the Γ-Y direction are estimated to be m^*α^_ΓY_ = 0.13 ± 0.02 *m*_*e*_ and m^*β^_ΓY_ = 0.20 ± 0.03 *m*_*e*_, which remain almost the same within the fitting error. As a result, the effective mass does not affect *PF* significantly.

## Conclusions

In summary, the electron band structure of a SnSe single crystal has been investigated using ARPES. The different cooling rate during the growth process leads to the overall multi-valley band shift of the electron band structure of SnSe, indicating that the cooling rate tunes the population of Sn vacancies and hence the charge carrier density, which is supported by first-principles calculations. Surprisingly, the shift of the electron band structure exactly follows the cooling rate dependence of the thermoelectric power factor of SnSe. These findings suggest a simple but efficient way to fabricate an intrinsic defect-induced high-efficiency thermoelectric phase of SnSe and provide a viable route toward the engineering of thermoelectric performance via the sample growth condition without an additional *ex-situ* process.

## Supplementary Information

**Additional file 1: Figure S1.** Comparison of core-level spectra for SnSe with or without the photoemission charging effect. **Figure S2.** ARPES E-k dispersions along the Γ-Y direction for SnSe single crystals with different growth cooling rates.

## Abbreviations

VBM: Valence band maximum
PF: Power factor
ARPES: Angle-resolved photoemission spectroscopy
DFT: Density functional theory
PBE + mBJ: Perdew-Burke-Ernzerhof functional combined with modified Becke-Johnson potential
CPA: Coherent potential approximation
KKR: Korringa-Kohn-Rostoker
