# Supplementary material for: Enhanced thermoelectric performance of SnSe by controlled vacancy population
Source: Nano Converg. 2023 Jul 7;10:32. doi: 10.1186/s40580-023-00381-7 (PMC10328875; doi:10.1186/s40580-023-00381-7)
Supplement: Supplementary file 1 — Additional file 1: Figure S1. Comparison of core-level spectra for SnSe with or without the photoemission charging effect. Figure S2. ARPES E-k dispersions along the Γ-Y direction for SnSe single crystals with different growth cooling rates. [file 40580_2023_381_MOESM1_ESM.docx]

Additional file 1 for

**Enhanced thermoelectric performance of SnSe by controlled vacancy population**

Ji-Eun Lee^1,2,3,4^, Kyoo Kim^2,5,6^, Van Quang Nguyen^7^, Jinwoong Hwang^1,4,8^, Jonathan D. Denlinger^1^, Byung Il Min^6^, Sunglae Cho^7^, Hyejin Ryu^1,2,3*^, Choongyu Hwang^4*^, Sung-Kwan Mo^1*^

*^1^Advanced Light Source, Lawrence Berkeley National Laboratory, Berkeley, CA 94720, USA*

*^2^Max Planck-POSTECH/Hsinchu Center for Complex Phase Materials, Max Plank POSTECH/Korea Research Initiative (MPK), Gyeongbuk 37673, South Korea*

*^3^Center for Spintronics, Korea Institute of Science and Technology, Seoul 02792, South Korea*

*^4^Department of Physics, Pusan National University, Busan 46241, South Korea*

*^5^Korea Atomic Energy Research Institute, Daejeon 34057, South Korea*

*^6^Department of Physics, Pohang University of Science and Technology (POSTECH), Pohang 37673, South Korea*

*^7^Department of Physics and Energy Harvest-Storage Research Center, University of Ulsan, Ulsan 44610, South Korea*

*^8^Department of Physics, Kangwon National University, Chuncheon 24341, South Korea*

*Corresponding authors: Hyejin Ryu, Choongyu Hwang, Sung-Kwan Mo

Tel.: +1-510-495-2903

*Email address: [*hryu@kist.re.kr*](mailto:hryu@kist.re.kr)*, ckhwang@pusan.ac.kr,* [*skmo@lbl.gov*](mailto:skmo@lbl.gov)

**Examination of the charging effect in SnSe by photon flux dependence**

To test the charging effect in photoemission process, we performed charging tests on all the samples prior to the ARPES measurement by taking core-level spectra with different incident photon flux. In Figure S1, core-level photoemission spectra were taken at 18 K, on the samples with a crystal growth cooling rate of 0.5 K/h. When the charging effect exists (Figure S1 a and b), we observe all the peaks shift towards higher binding energy with increasing photon flux. When the charging effect is absent in Figure S1 c and d, no peak shift was observed. The samples with the charging effect were discarded and all the data presented in this work is free of the charging effect.


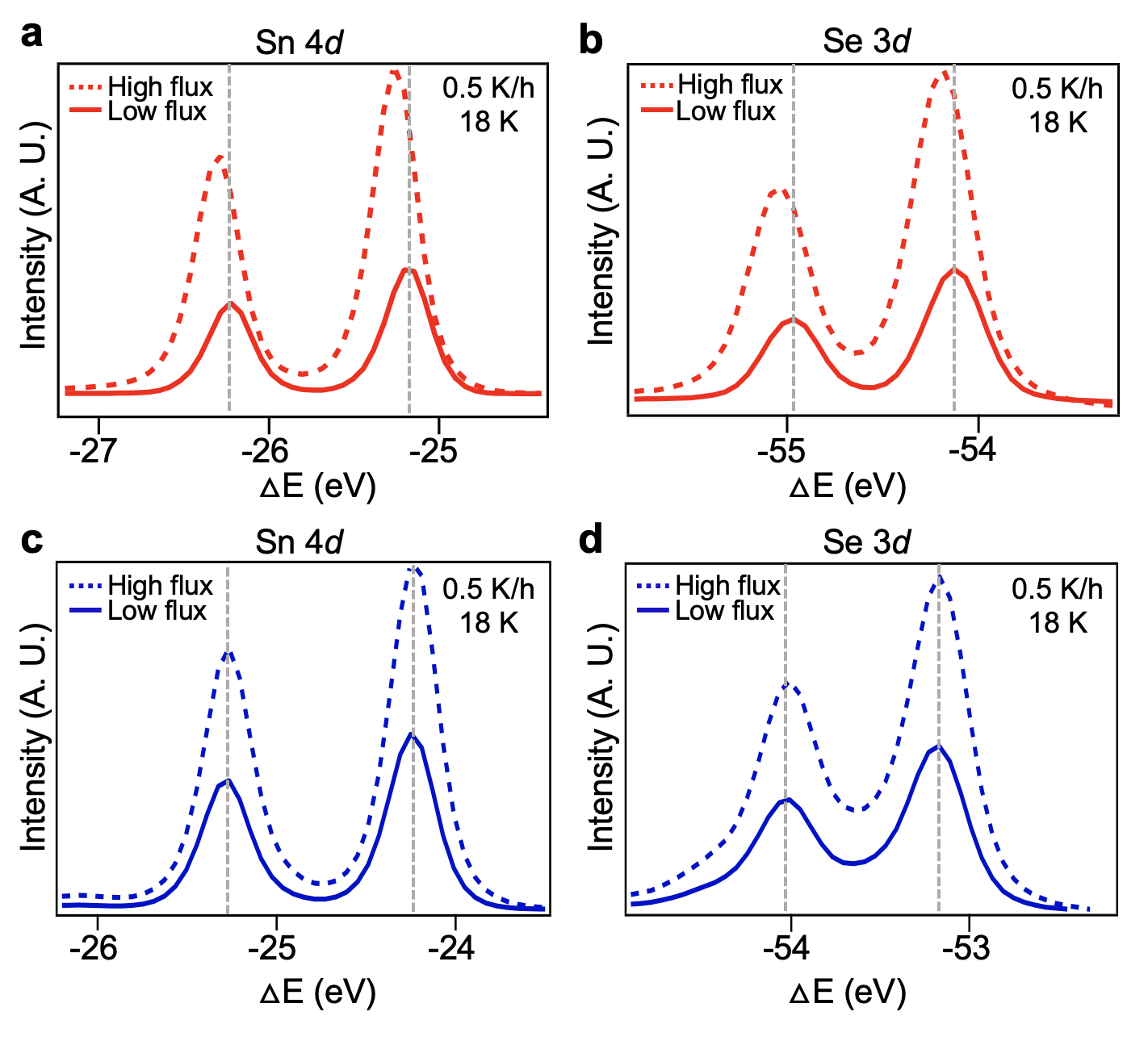


**Figure S1. Comparison of core-level spectra for SnSe with or without the photoemission charging effect.** The spectra show Sn 4d and Se 3d peaks (a, b) with and (c, d) without the charging effect. ΔE is the energy relative to the VBM of a 0.5 K/h sample. To improve visibility of the peak shift, gray dashed lines are included as a guideline. All the data were taken at 18 K for the samples with a crystal growth cooling rate of 0.5 K/h.

**ARPES *E-k* dispersions for the SnSe samples with different crystal growth cooling rates**

Figure S2 (a) shows ARPES *E*-*k* dispersions for the samples with a crystal growth cooling rate of 0.5 K/h, 1 K/h, 3 K/h, 4 K/h, and 5 K/h along the Γ-Y direction, together with corresponding second-derivative data in Figure S2 (b). The VBM near the Y point and parabolic bands at the Γ point evidently move downwards as the growth cooling rate increases.

**Figure S2. ARPES E-k dispersions along the Γ-Y direction for SnSe single crystals with different growth cooling rates.** (a) ARPES band dispersions for the samples with a crystal growth cooling rate of 0.5 K/h, 1 K/h, 3 K/h, 4 K/h, and 5 K/h. ΔE is the energy relative to the VBM of the 0.5 K/h sample. (b) Corresponding second derivative bands. All the data were obtained at a photon energy of 60 eV, except for the 0.5 K/h sample measured at 72 eV.
